# Exploring comorbidity and pharmacological treatment patterns in psoriasis - a retrospective population-based cross-sectional study

**DOI:** 10.1177/26335565231212336

**Published:** 2023-10-30

**Authors:** Nelly Hamgård, Albert Duvetorp, Anna Hägg, Sandra Jerkovic Gullin, Oliver Seifert

**Affiliations:** 1 Division of Cell Biology, Department of Biomedical and Clinical Sciences, 568027 Linköping University, Linköping, Sweden; 2 Department of Dermatology and Venereology, Lund University, Skane University Hospital, Malmö, Sweden; 3 Department of Dermatology and Venereology, 4160 Värnamo Hospital, Region Jönköping County, Sweden

**Keywords:** Psoriasis, comorbidity, drug dispensation

## Abstract

**Background:**

Individuals with psoriasis face an increased risk of suffering from various comorbid conditions. However, there haven´t been any studies conducted in Sweden to examine the frequency of comorbidities and the corresponding treatment for these conditions among psoriasis patients.

**Methods:**

The Cosmic electronic medical record represents a comprehensive repository of medical information including all residents of Region Jönköping. To conduct a population-based retrospective cross-sectional study, all individuals diagnosed with psoriasis between April 9, 2008 and July 1, 2016, were identified via the electronic medical records using ICD-10 codes. Data on comorbidity and dispensation of prescribed drugs against these comorbid conditions were analyzed.

**Results:**

During the study period, 1.7% of the population (4,587 individuals) were diagnosed with psoriasis, with 74.3% of cases classified as mild to moderate psoriasis and 25.7% as severe. The remaining 268,949 individuals did not receive a psoriasis diagnosis. The study showed that psoriasis patients had higher odds of experiencing the majority of the comorbid conditions examined. Arthritis other than psoriasis arthritis was found to be the most prevalent comorbid diagnosis among psoriasis patients (adjusted OR 7.2, CI 95% 6.4-8.2, p < 0.001), followed by obesity (OR 2.4, CI 95% 1.9-3.1, p < 0,001). There was no significant difference in drug prescription for comorbid diseases between patients with psoriasis and patients without psoriasis except for arthritis and smoking cessation treatment.

**Conclusions:**

Individuals with psoriasis are more susceptible to experiencing multiple comorbid conditions compared to the general population, particularly those with severe psoriasis. There is no evidence of undertreatment of comorbidity except for arthritis among psoriasis patients.

## Background

Psoriasis is an inflammatory skin disease that affects approximately 60 million adults and children worldwide.^1^ There is significant variation in the prevalence across populations worldwide, and many areas lack available data. Epidemiological data is only available for 20 out of the 194 member states of the World Health Organization (WHO).^2^ The global range of prevalence is from 0.1% in Asia to 2.7% in Europe.^3-5^ Recent epidemiological studies from Canada ^6^, Denmark^7^ and the UK^8^ showed a psoriasis prevalence of 2.5, 2.2 and 2.8%, respectively. A multicenter study revealed that among 22,060 individuals questioned, the self-reported prevalence of psoriasis was 9.7% in Norway, 9.4% in Sweden and 9.2% in Denmark. Psoriasis manifests without sex bias and emerges on average at the age of 33 with a bimodal peak of onset at 16-22 years and 55-60 years.^9^ Psoriasis can lead to both physical and psychological distress, often resulting in reduced quality of life.^10,11^

Psoriasis is a heterogeneous inflammatory skin disease characterized by a range of symptoms. The most prevalent type is plaque psoriasis, which causes distinct red scaly plaques and accounts for almost 90% of all cases.^9^ The severity of psoriasis varies widely, with some patients experiencing lesions covering their entire skin surface while others only have single patches. Approximately 30% of individuals with psoriasis develop joint inflammation, known as psoriatic arthritis (PsA)^12,13^ which can result in debilitating conditions. However, it is important to note that some patients with psoriasis may develop other types of arthritis besides PsA, such as rheumatoid arthritis (RA).^14,15^

Psoriasis can be categorized as mild, moderate or severe. The majority of individuals with psoriasis have mild to moderate symptoms that can be controlled through outpatient care and treated with topical therapy. However, approximately 20% of individuals have severe disease that requires specialized care and systemic treatment.^16^

Traditionally considered a dermatological disorder, psoriasis is now acknowledged as a chronic, systemic and immune-mediated inflammatory disease with a complex pathogenesis that involves various genetic, environmental and immunological factors.^17^ Moreover, psoriasis has been linked to several comorbid conditions, including diabetes mellitus, hypertension, obesity, hyperlipidemia, Crohn´s disease, ulcerative colitis, depression, and cardiovascular diseases such as stroke and myocardial infarction.^18^ Furthermore, psoriasis patients exhibit a high prevalence of smoking and alcohol consumption. ^19,20^ Comorbidity rates are particularly elevated in cases of moderate to severepsoriasis.^21^ The elevated likelihood of comorbidity in psoriasis patients leads to increased mortality in this population.^22^

In Sweden, studies have been conducted to determine the prevalence of psoriasis regionally.^23^ However, few Swedish studies have investigated comorbidity in patients with psoriasis. One recent study published by Hajiebrahimi et al. demonstrated a higher prevalence of cardiovascular disease and diabetes mellitus among individuals with psoriasis compared to the general population.^24^ Additionally, another study revealed an elevated risk of suffering from pharmacologically treated depression in psoriasis patients.^25^

Despite abundant data highlighting increased comorbidity in patients with psoriasis, no previous studies have investigated whether these comorbidities are being treated pharmacologically. Moreover, many of the above described studies on comorbidity rely solely on ICD-codes in medical journals without validating the accuracy of these codes. To address this research gap, the present study aimed to explore and provide insights into patterns of comorbidity and the corresponding medication. Similarly, we were interested in analyzing rheumatoid arthritis and other types of arthritis beyond PsA, considering that these conditions are less well investigated.

The objective of this study was to assess the prevalence of psoriasis in Region Jönköping, compare comorbidity rates and lifestyle factors such as smoking and alcohol intake between patients with and without psoriasis, and investigate if this correlation is related to the severity of psoriasis. Moreover, we intended to assess the dispensing of prescribed drugs for these comorbid conditions to verify the accuracy of comorbidity ICD-10 coding in medical records and investigate whether psoriasis patients receive adequate treatment for their comorbid conditions.

## Methodology

### Study design

The study method has been previously prescribed.^26^ Briefly, in 2008, the electronic medical record system Cosmic has been implemented in the healthcare system of Region Jönköping. This system covers the entire population of Jönköping, including those who have not received healthcare services. Cosmic records contain healthcare contacts, whether in outpatient and inpatient care. To conduct a retrospective population-based cross-sectional study on comorbidity among patients with psoriasis in Region Jönköping, all individuals diagnosed with psoriasis between 04/09/2008 and 07/01/2016 were identified using the Cosmic system. As of 01/01/2016, the population of the region was 341,845. The study included only individuals aged 18 and above (n=273,536) as diagnosing psoriasis in children can be more challenging.^27^ The study was approved by the Regional Ethical Review Board in Linköping, Sweden, diary number 2014/481-31 and 2015/416-32. This study involved the secondary analysis of already existing anonymous data and the Institutional Review Board deemed the study to pose minimal risk, hence, the need for obtaining consent was waived.

### Study population

All residents in Region Jönköping on 01/01/2016 aged 18 years and above were included in the study. To identify patients with psoriasis, the ICD-10 code L40 was searched in Cosmic. In order for the diagnosis to be valid, patients were required to have been diagnosed by a dermatologist at least once during the study period, or to have received the diagnosis from another clinic or primary care provider at least twice along with receiving targeted topical treatment (steroids group III-IV, calcipotriol, betnovate in combination with calcipotriol), or systemic treatment (Methotrexate, Cyclosporine, Acitretin, Apremilast, Dimethyl fumarate or biological drugs). Patients who were treated with systemic medication at least once during the study period were categorized as having severe psoriasis. The group of patients without psoriasis consisted of all residents in Region Jönköping aged 18 years and above who did not have the ICD-10 code L40. Age and sex information of the study participants was collected.

### Assessments

The primary endpoint of the study was the odds of comorbidity and lifestyle factors and possible treatment of these conditions among patients with psoriasis in Region Jönköping. The study analyzed the comorbidity reported in Cosmic for these patients. Table 1 provides the ICD-10 codes used for diagnosing these conditions. Notably, PsA was not the focus of this analysis. This study encompassed rheumatoid arthritis and other types of arthritis classified under ICD-10 codes M05 and M06, as outlined in Table 1.

**Table 1.** International Statistical Classification of Disease and Related Health Problems (ICD-10) used to identify comorbidity and lifestyle factors.

ICD-10	Disease
E10	Type 1 diabetes mellitus
E11	Type 2 diabetes mellitus
E66	Obesity
E78	Disorders of lipoprotein metabolism and other lipidemias
F10	Mental and behavioral disorders due to use of alcohol
F17	Nicotine dependence
F32	Depressive episode
F33	Recurrent depressive disorder
G35	Multiple sclerosis
I10	Essential (primary) hypertension
I20	Angina pectoris
K50	Crohn disease [regional enteritis]
M05	Rheumatoid arthritis with rheumatoid factor
M06	Other rheumatoid arthritis

To validate the accuracy of both psoriasis and comorbidity diagnoses, the study linked the prescription and dispensation of drugs for patients with and without psoriasis to the corresponding ICD-10 code. The National Board of Health and Welfare's drug register, established in July 2005, provided information on drug treatment, ATC code, social security number and all drugs that were collected against a prescription at a pharmacy. This data was integrated with information from Cosmic. Table 2 presents the ATC codes utilized in the study.

**Table 2.** Anatomical Therapeutic Chemical Classification System (ATC) codes used to identify drugs.

Concomitant drug	ATC code (ICD-10 code for related comorbidity)
Medication for inflammatory bowel disease	A07E: Intestinal anti-inflammatory agents (K50)
Medication for obesity	A08A: anti-obesity preparations, excl. diet products (E66)
Diabetes medication	A10: Drugs used in diabetes (E10 and E11)
Nitrates (vasodilators)	C01D: Vasodilators used in cardiac diseases (I20)
Diuretics	C03: Diuretics (I10)
Beta blockers	C07: Beta blocking agents (I10)
Calcium channel blockers	C08: Calcium channel blockers (I10)
ACE inhibitors and angiotensin II receptor blockers	C09: Agents acting on the renin-angiotensin system (I10)
Lipid modifying agents	C10: Lipid modifying agents (E78)
Topical calcipotriol, combinations with betamethasone or ichthammol	D05A: Antipsoriatics for topical use (L40)
Dimethyl fumarate or acitretin	D05B: Antipsoriatics for systemic use (L40)
Topical corticosteroids class III or IV	D07AC: Corticosteroids potent, group III (L40) D07AD: Corticosteroids, very potent, group IV (L40)
Methotrexate	L01BA01: Methotrexate (L40, M05, M06)
Immunosuppressive drugs	L04AA: Selective immunosuppressants, exclusive Apremilast (G35, K50, M05, M06) L04AX: Other immunosuppressants, exclusive Methotrexate and Dimethyl fumarate (G35, M05, M06, K50) M01C: Specific antirheumatic agents (M05, M06)
Apremilast	L04AA32: Apremilast (L40)
TNF-α inhibitors	L04AB: Tumor necrosis factor alpha (TNF-α) inhibitors (K50, L40, M05, M06)
Interleukin inhibitors	L04AC: Interleukin inhibitors (L40, M05, M06)
Calcineurin inhibitors	L04AD: Calcineurin inhibitors (L40)
Antirheumatic products, non-steroids	M01A: Anti-inflammatory and antirheumatic products, non-steroids (M05, M06)
Topical anti-inflammatory products	M02: Topical products for joint and muscular pain (M05, M06)
Peroral steroids	H02AB: corticosteroids for systemic use, plain (G35, K50, M05, M06)
Antidepressants	N06A: Antidepressants (F32, F33))
Drugs used in nicotine and alcohol dependence	N07BA: Drugs used in nicotine dependence (F17) N07BB: Drugs used in alcohol dependence (F10)

### Statistical analysis

Statistical analysis was conducted using SPSS version 27.0. The odds ratio (OR) with a 95% confidence interval (CI) was calculated through multinomial logistic regression to estimate the odds of comorbidity and to compare the treatment frequency for comorbid diseases. This multinomial regression had a 3-category outcome: 1) no comorbidity, 2) comorbidity with drug treatment and 3) comorbidity without drug treatment. For each comorbidity and lifestyle factor, a single statistical model was employed , adjusting for age and sex. In addition, a multivariate model with the same outcome was used adjusted for age, sex and for all comorbidities and lifestyle factors.

## Results

### Demographic data

Over the course of the study, 4,587 patients were diagnosed with psoriasis, which accounted for 1.7% of the population. Of those diagnosed, 2,282 were males (49.7%) and 2,305 were females (50.3%). The age range of the patients was between 18-103 years with a mean age of 57.4 ± 17.3 years (mean ± standard deviation (SD)). The largest age group of patients fell between 61 and 70 years old, accounting for 23.9% of all psoriasis patients.

The group of patients without psoriasis included 268,949. The sex distribution of this group was equal, with 50% males and 50% females. The age range of individuals in the group of patients without psoriasis was between 18 and 105 years with a mean age of 49.7 ± 19.5 (mean ± SD). The largest proportion of individuals in this group (21.2%) were under the age of 30 (Table 3).

**Table 3.** Demographic data for all patients with psoriasis and patients without psoriasis.

Variable	Patients with psoriasis (n=4,587)		Patients wihout Psoriasis (n=268,949)	
	n	%	n	%
Sex				
Male	2,282	49.7	134,560	50.0
Female	2,305	50.3	134,389	50.0
Age				
Mean value (±SD)	57.4 ± 17.3		49.7 ± 19.5	
Interval	18–103		18–105	
<= 30	411	9.0	57,908	21.2
31-40	433	9.4	39,641	14.5
41-50	707	15.4	46,018	16.8
51-60	814	17.7	42,107	15.4
61-70	1,094	23.9	41,088	15.0
71-80	781	17.0	28,898	10.6
81-90	310	6.8	14,811	5.4
91+	37	0.8	3,065	1.1

Out of all psoriasis patients, 3,407 (74.3%) had mild to moderate psoriasis, of which 1,677 (49.5%) were males and 1,730 (50.8%) were females. In contrast, 1,180 (25.7%) had severe psoriasis, as indicated by their use of systemic therapy. Among these patients, 605 (51.3%) were males and 575 (48.7%) were females (Table 4).

**Table 4.** Demographic data for patients with mild to moderate and severe psoriasis.

Variable	Mild to moderate psoriasis (n=3,407)		Severe psoriasis (n=1,180)	
	n	%	n	%
Sex				
Male	1,677	49.2	605	51.3
Female	1,730	50.8	575	48.7
Age				
Mean value (±SD)	57.5 ± 17.9		57.07 ± 15.26	
Interval	18–103		18–97	
<= 30	339	10.0	72	6.1
31-40	335	9.8	98	8.3
41-50	493	14.5	214	18.1
51-60	552	16.2	262	22.2
61-70	793	23.3	301	25.5
71-80	598	17.6	183	15.5
81-90	264	7.7	46	3.9
91+	33	1.0	4	0.3

In the analysis of comorbidities and lifestyle factors, hypertension, hyperlipidemia and depression emerged as the three most frequently diagnosed conditions in both patients with or without psoriasis (Supplemental Table 1). The highest proportion of patients, with our without psoriasis, not receiving treatment for a specific comorbidity and lifestyle factor was observed for nicotine dependence, obesity and alcohol dependence.

### Comorbidity in all psoriasis patients

We conducted a multinomial regression analysis with a 3-category outcome to assess the difference between patients with psoriasis and those without it. This analysis compared the following three outcomes: 1. Patients with no comorbidity (co-). 2. Patients with comorbidity (co+) receiving treatment (treat+) and 3. Patients with comorbidity (co+) not receiving treatment (treat-). These comparisons were made with adjustment for the influence of age and sex on the odds. In general, the study demonstrated a heightened likelihood of comorbidity in individuals with psoriasis irrespective of treatment across the majority of scrutinized diagnoses (Table 5).

**Table 5.** Comorbidity and and related drug treatment in psoriasis patients compared to patients without psoriasis (single statistical model adjusting for age and sex).

Comorbidity	All psoriasis adj. OR (95% CI)	sign. level	Mild/moderate psoriasis adj. OR (95% CI)	sign. level	Severe psoriasis adj. OR (95% CI)	sign. level
	*crude OR*		*crude OR*		*crude OR*	
Arthritis (Ar)	Ref: Ar-					
Ar+ and treat+	7.2 (6.4-8.2) 8.5 (7.5-9.5)	***	0.9 (0.6-1.3) 1.1 (0.8-1.5)	ns	33.4 (28.9-38.6) 35.2 (30.7-40.5)	***
Ar+ and treat-	12.3 (8.8-17.2) 14.1 (10.1-19.7)	***	14.8 (10.5-20.8) 17.4 (12.4-24.5)	***	1.2 (0.2-8.9) 1.3 (0.2-9.4)	ns
Ref: Ar+ and treat-						
Ar+ and treat+	0.6 (0.4-0.8) 0.6 (0.4-0.9)	**	0.06 (0.04-0.09) 0.06 (0.04-0.1)	***	26.9 (3.8-192.7) 26.8 (3.8-192.1)	***
Nicotine dependence (Nd)	Ref: Nd-					
Nd+ and treat+	2.8 (2.2-3.4) 3.2 (2.6-3.9)	***	2.5 (1.9-3.1) 2.9 (2.3-3.6)	***	3.5 (2.5-4.9) 3.9 (2.8-5.5)	***
Nd+ and treat-	2.0 (1.8-2.3) 2.4 (2.1-2.7)	***	1.9 (1.6-2.2) 2.2 (1.9-2.5)	***	2.4 (1.9-3.1) 2.8 (2.2-3.5)	***
Ref: Nd+ and treat-						
Nd+ and treat+	1.4 (1.1-1.7) 1.4 (1.1-1.7)	**	1.3 (1.0-1.8) 1.3 (1.0-1.7)	*	1.4 (0.9-2.2) 1.4 (0.9-2.2)	ns
Diabetes (DM)	Ref: DM-					
DM+ and treat+	1.9 (1.7-2.0) 2.4 (1.6-2.6)	***	1.8 (1.6-2.0) 2.3 (2.1-2.6)	***	2.0 (1.7-2.4) 2.3 (1.9-2.8)	***
DM+ and treat-	1.6 (1.3-2.0) 2.0 (1.6-2.6)	***	1.4 (1.1-1.9) 1.9 (1.5-2.5)	*	2.1 (1.4-3.2) 2.3 (1.5-3.6)	***
Ref: DM+ and treat-						
DM+ and treat+	1.2 (0.9-1.5) 1.2 (0.9-1.5)	ns	1.3 (0.9-1.7) 1.2 (0.9-1.6)	ns	0.9 (0.6-1.5) 0.9 (0.6-1.6)	ns
Depression (Dep)	Ref: Dep-					
Dep+ and treat+	1.6 (1.4-1.7) 1.7 (1.5-1.8)	***	1.5 (1.3-1.6) 1.6 (1.4-1.7)	***	1.8 (1.5-2.1) 1.9 (1.6-2.2)	***
Dep+ and treat-	1.5 (1.2-1.9) 1.3 (1.0-1.7)	***	1.5 (1.2-2.1) 1.4 (1.0-1.8)	**	1.4 (0.8-2.3) 1.2 (0.7-2.0)	ns
Ref: Dep+ and treat-						
Dep+ and treat+	1.0 (0.8-1.3) 1.3 (0.9-1.6)	ns	0.9 (0.7-1.3) 1.2 (0.9-1.5)	ns	1.3 (0.8-2.2) 1.6 (0.9-2.7)	ns
Alcohol dependence (Ad)	Ref: Ad-					
Ad+ and treat+	1.6 (1.3-2.1) 1.8 (1.4-2.3)	***	1.5 (1.1-2.1) 1.7 (1.2-2.2)	**	1.9 (1.2-2.9) 2.0 (1.3-3.2)	**
Ad+ and treat-	2.0 (1.6-2.5) 2.0 (1.6-2.5)	***	2.0 (1.6-2.6) 1.9 (1.6-2.6)	***	2.0 (1.3-3.0) 2.0 (1.3-3.1)	**
Ref: Ad+ and treat-						
Ad+ and treat+	0.8 (0.6-1.1) 0.9 (0.6-1.2)	ns	0.8 (0.5-1.1) 0.8 (0.6-1.2)	ns	0.9 (0.5-1.7) 1.0 (0.5-1.9)	ns
Obesity (Ob)	Ref: Ob-					
Ob+ and treat+	2.4 (1.9-3.1) 2.6 (1.9-3.3)	***	2.1 (1.5-2.9) 2.2 (1.6-3.0)	***	3.4 (2.2-5.1) 3.4 (2.3-5.3)	***
Ob+ and treat-	2.0 (1.9-2.2) 2.3 (2.1-2.5)	***	1.9 (1.7-2.1) 2.1 (1.9-2.4)	***	2.4 (2.0-2.8) 2.7 (2.2-3.1)	***
Ref: Ob+ and treat-						
Ob+ and treat+	1.2 (0.9-1.6) 1.1 (0.9-1.5)	ns	1.1 (0.8-1.5) 1.0 (0.8-1.4)	ns	1.4 (0.9-2.2) 1.3 (0.8-2.0)	ns
Crohn´s disease (Cd)	Ref: Cd-					
Cd+ and treat+	1.5 (1.0-2.3) 1.5 (1.0-2.3)	*	0.8 (0.5-1.6) 0.8 (0.5-1.6)	ns	3.5 (2.1-5.9) 3.5 (2.0-5.9)	***
Cd+ and treat-	1.3 (0.5-3.4) 1.4 (0.5-3.8)	ns	1.3 (0.4-4.0) 1.4 (0.5-4.5)	ns	1.3 (0.2-8.9) 1.4 (0.2-9.9)	ns
Ref: CD+ and treat-						
Cd+ and treat+	1.2 (0.4-3.5) 1.1 (0.4-3.1)	ns	0.7 (0.2-2.4) 0.6 (0.2-2.2)	ns	2.7 (0.4-21.2) 2.5 (0.3-19.1)	ns
Angina pectoris (Ap)	Ref: Ap-					
Ap+ and treat+	1.5 (1.3-1.7) 1.9 (1.7-2.2)	***	1.4 (1.2-1.6) 1.9 (1.6-2.2)	***	1.8 (1.4-2.4) 1.9 (1.5-2.5)	***
Ap+ and treat-	1.9 (1.4-2.6) 2.4 (1.7-3.3)	***	1.6 (1.1-2.4) 2.1 (1.4-3.1)	***	2.8 (1.6-4.8) 3.3 (1.9-5.6)	***
Ref: Ap+ and treat-						
Ap+ and treat+	0.8 (0.6-1.1) 0.8 (0.6-1.1)	ns	0.9 (0.6-1.3) 0.9 (0.6-1.4)	ns	0.7 (0.4-1.2) 0.6 (0.3-1.1)	ns
Hypertension (Ht)	Ref: Ht-					
Ht+ and treat+	1.8 (1.7-1.9) 2.3 (2.2-2.5)	***	1.7 (1.5-1.8) 2.2 (2.1-2.4)	***	2.2 (1.9-2.5) 2.5 (2.2-2.8)	***
Ht+ and treat-	1.6 (1.2-2.2) 2.1 (1.6-2.8)	***	1.4 (0.9-2.0) 1.8 (1.3-2.6)	ns	2.4 (1.5-3.9) 2.9 (1.8-4.7)	***
Ref: Ht+ and treat-						
Ht+ and treat+	1.1 (0.8-1.5) 1.1 (0.8-1.5)	ns	1.2 (0.8-1.7) 1.3 (0.9-1.8)	ns	0.9 (0.6-1.5) 0.9 (0.5-1.4)	ns
Hyperlipidemia (Hl)	Ref: Hl-					
Hl+ and treat+	1.7 (1.6-1.9) 2.2 (2.1-2.4)	***	1.7 (1.6-1.9) 2.2 (2.1-2.4)	***	1.8 (1.5-2.0) 2.0 (1.8-2.4)	***
Hl+ and treat-	1.6 (1.1-2.1) 1.9 (1.4-2.6)	**	1.3 (0.9-1.9) 1.6 (1.1-2.4)	ns	2.2 (1.3-3.7) 2.6 (1.6-4.4)	**
Ref: HL+ and treat-						
Hl + and treat+	1.1 (0.8-1.5) 1.2 (0.9-1.6)	ns	1.3 (0.9-1.9) 1.4 (0.9-2.1)	ns	0.8 (0.5-1.3) 0.8 (0.5-1.3)	ns
Multiple sclerosis (MS)	Ref: MS-					
MS+ and treat+	1.7 (0.8-3.7) 1.8 (0.9-3.9)	ns	2.0 (0.9-4.5) 2.1 (0.9-4.8)	ns	1.0 (0.1-7.0) 1.0 (0.1-7.2)	ns
MS+ and treat-	1.8 (1.1-3.1) 2.0 (1.2-3.4)	*	2.3 (1.3-3.9) 2.5 (1.4-4.3)	**	0.5 (0.1-3.5) 0.5 (0.1-3.8)	ns
Ref: MS+ and treat-						
MS+ and treat+	1.0 (0.4-2.4) 0.9 (0.4-2.4)	ns	0.9 (0.3-2.4) 0.9 (0.3-2.3)	ns	1.9 (0.1-31.3) 1.9 (0.1-30.2)	ns

For each comorbidity and lifestyle factor a multinomial regression with a 3-category outcome was performed: 1) no comorbidity (co-), 2) comorbidity (co+) with treatment (treat+) and 3) comorbidity (co+) without treatment (treat-). All three outcomes were compared to each other. (Ref = reference for comparison in the regression analysis, adj. OR= odds ratio adjusted for age and sex with 95% confidence interval (CI), sign. level = significance level= * p < 0.05; ** p < 0.01; *** p < 0.001, ns = no significant difference).

Rheumatoid arthritis and other arthritis identified by ICD-10 code M05 and M06 (according to Table 1) exhibited the strongest association among individuals with psoriasis, as indicated by an OR of 7.2 (95% CI 6.4-8.2) for those having received arthritis treatment and an OR of 12.3 (95% CI 8.8-17.2) for those not receiving arthritis treatment. Moreover, psoriasis patients were found to have a higher susceptibility to obesity (OR 2.4, 95% CI 1.9-3.1 with treatment and OR 2.0 (CI 1.9-2.2) without treatment) and were more likely to smoke than patients without psoriasis, with an OR of 2.8 (CI 2.2-3.4) for those with treatment and 2.0 (CI1.8-2.3) for those without treatment.

A discrepancy was identified regarding treatment in multiple sclerosis (MS) and Crohn´s disease. There was no significant difference between patients with psoriasis and those without it having Crohn´s disease without treatment. The opposite was true for MS. Here, we observed no statistical difference between patients with psoriasis and those without it having treatment for MS . However, a significant association was found when analyzing MS without treatment, with patients having psoriasis demonstrating a significant association compared to those without psoriasis.

To explore the potential influence of comorbidities and lifestyle factors on the three aforementioned outcomes, we conducted a multinomial regression now incorporating all comorbidities and lifestyle factors as explanatory variables (multivariable) in addition to age and sex. The results are presented in Table 6. Adjusting for all comorbidities and lifestyle factors did not change the results for arthritis, depression, obesity, hypertension and MS.

**Table 6.** Comorbidity and and related drug treatment in psoriasis patients compared to patients without psoriasis (multivariate model adjusted for age, sex and for all comorbidities and lifestyle factors).

Comorbidity	All psoriasis adj. OR (95% CI)	sign. level	Mild/moderate psoriasis adj. OR (95% CI)	sign. level	Severe psoriasis adj. OR (95% CI)	sign. level
Arthritis (Ar)	Ref: Ar-					
Ar+ and treat+	6.8 (6.1-7.8)	***	0.8 (0.6-1.2)	ns	31.1 (26.9-36.0)	***
Ar+ and treat-	11.6 (8.2-16.3)	***	14.0 (9.9-19.8)	***	1.1 (0.2-7.9)	ns
Ref: Ar+ and treat-						
Ar+ and treat+	0.6 (0.4-0.9)	**	0.06 (0.04-0.1)	***	27.9 (3.9-199.9)	***
Nicotine dependence (Nd)	Ref: Nd-					
Nd+ and treat+	2.0 (1.7-2.5)	***	1.9 (1.5-2.5)	***	2.3 (1.6-3.2)	***
Nd+ and treat-	1.6 (1.4-1.9)	***	1.6 (1.3-1.8)	***	1.8 (1.4-2.3)	***
Ref: Nd+ and treat-						
Nd+ and treat+	1.3 (0.9-1.6)	ns	1.2 (0.9-1.6)	ns	1.2 (0.8-1.9)	ns
Diabetes (DM)	Ref: DM-					
DM+ and treat+	1.3 (1.2-1.5)	***	1.3 (1.2-1.5)	***	1.3 (1.1-1.6)	*
DM+ and treat-	1.2 (0.9-1.6)	ns	1.1 (0.8-1.5)	ns	1.6 (1.1-2.5)	*
Ref: DM+ and treat-						
DM+ and treat+	1.1 (0.8-1.4)	ns	1.2 (0.9-1.6)	ns	0.8 (0.5-1.3)	ns
Depression (Dep)	Ref: Dep-					
Dep+ and treat+	1.3 (1.2-1.5)	***	1.3 (1.2-1.4)	***	1.5 (1.3-1.7)	***
Dep+ and treat-	1.4 (1.1-1.8)	*	1.4 (1.1-1.9)	*	1.2 (0.7-2.0)	ns
Ref: Dep+ and treat-						
Dep+ and treat+	0.9 (0.8-1.3)	ns	0.9 (0.7-1.2)	ns	1.2 (0.7-2.1)	ns
Alcohol dependence (Ad)	Ref: Ad-					
Ad+ and treat+	1.2 (0.9-1.6)	ns	1.2 (0.9-1.6)	ns	1.3 (0.8-2.1)	ns
Ad+ and treat-	1.6 (1.3-2.0)	***	1.6 (1.3-2.1)	***	1.4 (0.9-2.2)	ns
Ref: Ad+ and treat-						
Ad+ and treat+	0.8 (0.6-1.1)	ns	0.7 (0.5-1.1)	ns	0.9 (0.5-1.7)	ns
Obesity (Ob)	Ref: Ob-					
Ob+ and treat+	1.9 (1.4-2.4)	***	1.6 (1.2-2.2)	**	2.5 (1.6-3.9)	***
Ob+ and treat-	1.6 (1.4-1.7)	***	1.5 (1.3-1.7)	***	1.8 (1.5-2.1)	***
Ref: Ob+ and treat-						
Ob+ and treat+	1.2 (0.9-1.5)	ns	1.1 (0.8-1.5)	ns	1.4 (0.9-2.2)	ns
Crohn disease (Cd)	Ref: Cd-					
Cd+ and treat+	1.3 (0.9-1.9)	ns	0.8 (0.4-1.5)	ns	2.5 (1.4-4.3)	***
Cd+ and treat-	1.3 (0.5-3.6)	ns	1.3 (0.4-4.0)	ns	1.4 (0.2-10.3)	ns
Ref: CD+ and treat-						
Cd+ and treat+	1.0 (0.3-2.9)	ns	0.6 (0.2-2.3)	ns	1.7 (0.2-13.5)	ns
Angina pectoris (Ap)	Ref: Ap-					
Ap+ and treat+	1.1 (0.9-1.3)	ns	1.1 (0.9-1.2)	ns	1.2 (0.9-1.6)	ns
Ap+ and treat-	1.5 (1.1-2.1)	*	1.3 (0.9-2.0)	ns	2.0 (1.2-3.6)	*
Ref: Ap+ and treat-						
Ap+ and treat+	0.7 (0.5-1.0)	ns	0.8 (0.5-1.2)	ns	0.6 (0.3-1.1)	ns
Hypertension (Ht)	Ref: Ht-					
Ht+ and treat+	1.4 (1.3-1.5)	***	1.3 (1.2-1.4)	***	1.7 (1.4-2.0)	***
Ht+ and treat-	1.4 (1.1-1.9)	*	1.2 (0.8-1.7)	ns	2.1 (1.3-3.5)	**
Ref: Ht+ and treat-						
Ht+ and treat+	0.9 (0.7-1.3)	ns	1.1 (0.8-1.6)	ns	0.8 (0.5-1.3)	ns
Hyperlipidemia (Hl)	Ref: Hl-					
Hl+ and treat+	1.2 (1.1-1.3)	***	1.3 (1.1-1.4)	***	1.1 (0.9-1.3)	ns
Hl+ and treat-	1.1 (0.8-1.6)	ns	1.0 (0.7-1.5)	ns	1.4 (0.8-2.5)	ns
Ref: HL+ and treat-						
Hl + and treat+	1.1 (0.8-1.5)	ns	1.2 (0.8-1.8)	ns	0.8 (0.5-1.3)	ns
Multiple sclerosis (MS)	Ref: MS-					
MS+ and treat+	1.7 (0.8-3.6)	ns	1.9 (0.9-4.4)	ns	0.9 (0.2-6.3)	ns
MS+ and treat-	2.0 (1.2-3.4)	*	2.4 (1.4-4.1)	**	0.7 (0.1-4.8)	ns
Ref: MS+ and treat-						
MS+ and treat+	0.8 (0.3-2.1)	ns	0.8 (0.3-2.2)	ns	1.3 (0.1-20.7)	ns

For each comorbidity and lifestyle factor a multinomial regression with a 3-category outcome was performed: 1) no comorbidity (co-), 2) comorbidity (co+) with treatment (treat+) and 3) comorbidity (co+) without treatment (treat-). All three outcomes were compared to each other. (Ref = reference for comparison in the regression analysis, OR= odds ratio adjusted for age and sex and for all comorbidities and lifestyle factors with 95% confidence interval (CI), sign. level = significance level = * p < 0.05; ** p < 0.01; *** p < 0.001, ns = no significant difference).

However, the multivariable regression model did not reveal significant differences between all patients with psoriasis receiving treatment for alcohol dependence, Crohn´s disease, and angina pectoris compared to patients without psoriasis.

### Comorbidity in severe psoriasis

Despite dividing the psoriasis patients into different severity categories, a significant difference persisted for most comorbid conditions compared to patients without psoriasis. In patients with severe psoriasis (Table 5) the most significant difference was observed for rheumatoid arthritis and other arthritis (OR 33.4, 95% CI 28.9-38.6), followed by Crohn's disease (OR 3.5, 95% CI 2.1-5.9), smoking (OR 3.5, 95% CI 2.5-4.9) and obesity (OR 3.4, 95% CI 2.2-5.1). Interestingly, among patients with severe psoriasis and arthritis, depression and Crohn´s disease without treatment for these comorbid conditions, no significant difference was observed compared to patients without psoriasis. In patients with severe psoriasis, no significant difference was seen regarding MS which could be attributed to the low number of cases observed in this group.

In contrast, the multivariable regression model did not reveal significant differences between patients with severe psoriasis receiving treatment for alcohol dependence and hyperlipidemia compared to patients without psoriasis (Table 6).

### Comorbidity in mild psoriasis

The strength of association of comorbidity was weaker in patients with mild psoriasis compared to all patients with psoriasis or those with severe disease (Table 5). However, the majority of the diagnoses investigated in the study was still more prevalent in patients with mild psoriasis than in patients without psoriasis . The most significant differences were observed in smoking (OR 2.5, 95% CI 1.9-3.1) and obesity (OR 2.1, 95% CI 1.5-2.9).

A significant difference for the diagnosis of MS and arthritis was noticed only for those not receiving treatment for these diseases while there was no significant difference between patients with mild psoriasis with a diagnosis of MS or arthritis and receiving treatment compared to patients without psoriasis not diagnosed with MS or arthritis.

On the contrary, a significant difference emerged in the diagnosis of hypertension and hyperlipidemia exclusively among individuals undergoing treatment for these diseases. There was no significant difference observed between patients with mild psoriasis who had been diagnosed with hypertension and hyperlipidemia and were not receiving treatment, when compared to patients without psoriasis not diagnosed with hypertension and hyperlipidemia.

No significant difference was seen regarding Crohn's disease.

Interestingly, the multivariable regression model did not reveal significant differences between patients with mild psoriasis receiving treatment for alcohol dependence, Crohn´s disease and angina pectoris compared to patients without psoriasis (Table 6).

### Comparison of treatment frequency

To determine whether patients with psoriasis receive treatment for comorbid diseases or lifestyle factors to the same extent as patients without psoriasis, a comparison was made between patients with comorbidity (co+) receiving treatment (treat+) and patients with comorbidity (co+) not receiving treatment (treat-) (Table 5)

In summary , our findings suggested that the likelihood of receiving treatment or not for the respective comorbidity is not dependent on having a diagnosis of psoriasis. When comparing the ORs for most diagnosis, we did not observe a significant difference. Nevertheless, we observed that for all patients with psoriasis and those with mild psoriasis, the likelihood of not receiving treatment for arthritis was higher compared to patients without psoriasis. Conversely, having severe psoriasis increased the likelihood of receiving arthritis treatment.

Regarding smoking, the odds of receiving treatment to quit smoking was significantly higher among all patients with psoriasis and those with mild psoriasis compared to patients without psoriasis. . This significant difference was not seen in the multivariable regression model (Table 6).

Regarding all other comorbidities and lifestyle factors, the multivariable regression model revealed the same results as the regression analysis only adjusting for age and sex (Table 6).

## Discussion

In the present study, the prevalence of psoriasis was found to be 1.7%, which is lower than the expected prevalence. The global prevalence of psoriasis in adults ranges from 0.5 and 11.4% ^2^ with estimates of 2-4% in the Western world.^5^ In Scandinavia, a registry study conducted in Denmark in 2017 showed a prevalence of 2.2%^7^ while a Norwegian population-based study in 2013 reported a prevalence of 11.4%, albeit based on self-reported psoriasis diagnosis.^28^ In a study conducted in Skåne, Sweden, where the psoriasis diagnosis was validated by reviewing medical journals, medical journal ICD codes were found to be correct in at least 81% of cases.^23^ The same study also reported a prevalence of 1.23% which is consistent with the findings of our study. The prevalence of psoriasis, as reported in this study, is likely underestimated rather than overestimated due to strict diagnostic criteria for psoriasis. The study identified no difference in prevalence between sexes, consistent with previous research, although conflicting data also exists.^5^ The majority of psoriasis patients, approximately 70-80%, have a mild form of the disease that can be treated with topical steroids^29^ consistent with the current study´s findings, where 74.3% of cases were classified as mild.

Psoriasis is a chronic inflammatory disease caused by immune dysfunctions, which increases the risk of developing several other ailments.^30^ Several coexisting diseases have recently been observed to create substantial “comorbidity trajectory networks” which offer insights into their timely onset based on a patient´s medical history.^31^ The most prevalent and significant comorbidity associated with psoriasis is psoriatic arthritis (PsA).^32^ However, the prevalence of PsA varies widely, ranging from 6 and 41%^33^ depending on factors such as diagnostic criteria, the diagnosing physician, epidemiology and the population under examination. A recent comprehensive meta-analysis suggest that approximately 25% of patients with psoriasis suffer from PsA.^34^ A study of psoriasis patients across 34 different skin clinics in Europe and North America revealed that 30% of them had PsA^35^ which corresponds to the prevalence observed in Nordic^12^ and a German study.^13^ Nevertheless, individuals with psoriasis may also develop other types of arthritis apart from PsA.

As described in Table 1, the aim of this study was to investigate the prevalence of arthritis diseases other than PsA among patients with psoriasis. PsA was not considered as a comorbid diagnosis in this study. Out of all the psoriasis patients who participated in our study, 6.9% had been diagnosed with “rheumatoid arthritis with rheumatoid factor” (ICD-10 code M05) or “other rheumatoid arthritis” (M06) and were receiving treatment. The prevalence of RA in the Western world among adults is estimated to be around 0.5-1% of adults^36^ a figure similar to that of our group of patients without psoriasis. The incidence of RA among patients with psoriasis is not well documented, but a retrospective observational study conducted in the US indicated that patients with psoriasis had a three times higher incidence of RA than controls which is in line with the present results.^37^

Distinguishing between RA and PsA can be challenging due to the many clinical similarities between the two conditions^38,39^ which can lead to diagnostic uncertainty. Additionally, joint problems may occur prior to the onset of visible psoriasis symptoms.^40^ Arthritis was the most frequently observed comorbidity among psoriasis patients in the study. However, the significance of this finding varied depending on the severity of psoriasis. Patients with mild psoriasis did not exhibit any significant differences in the prevalence of drug-treated arthritis but showed significantly increased odds when having untreated arthritis. These findings indicate that patients with mild psoriasis tend to receive arthritis diagnoses more frequently, primarily because of the common occurrence of unspecific joint pain (arthralgia) in this group, as compared to patients without psoriasis. Conversely, drug-treated arthritis showed the strongest association in individuals with severe psoriasis, but was not significant when not being treated. . However, these results must be interpreted with caution. The group of untreated arthritis and severe psoriasis patients included only a single individual, potentially contributing to inconclusive findings. In this study, methotrexate served as a proxy for severe psoriasis but may even be prescribed for arthritis in the group of mild to moderate psoriasis. In our database, there is no indication of the condition the drug is prescribed for. There could be an overlap of biologics prescribed for severe psoriasis, psoriasis arthritis or other arthritis. Nonetheless, these results suggest that physicians are attentive in diagnosing arthritis in patients with severe psoriasis and likely to treat this highly associated comorbidity.

We found that the most notable difference in relation to Crohn's disease was observed in patients with severe psoriasis, whereas no significant difference was observed in those with mild psoriasis. A Danish cohort study have demonstrated that Crohn’s disease has a strong association with severe psoriasis.^41^ A study published in 2022 by Schneeweiss et al. revealed an increased risk of Crohn´s disease (HR 1.2, 95%CI 1.03-1.46) in patients with psoriasis which is in line with our data ^42^. Other previous studies have shown an increased risk of inflammatory bowel disease in individuals with psoriasis with two meta-analyses reporting ORs of 1.70^43^ and 2.0^44^ for Crohn's disease. These ratios are slightly higher than what we observed for the entire psoriasis group. However, it should be noted that the severity of psoriasis was not reported in these meta-analyses, which may have affected the results. Notably, our findings revealed no significant difference when comparing patients with both psoriasis and untreated Crohn´s disease to those without psoriasis. These results are challenging to explain, but one might speculate that patients with psoriasis might experience more severe Crohn´s disease, requiring treatment.

The relationship between psoriasis and multiple sclerosis (MS) is a topic of debate, with conflicting results from previous research. However, more recent studies indicate a significant correlation between the two conditions.^45-47^ A meta-analysis shows an increased odds ratio of 1.29.^48^ A Danish study indicates that the risk of MS may be influenced by the severity of psoriasis.^45^ In our study, only two patients with severe psoriasis were diagnosed with MS, making it challenging to assess the lack of significance. Significant correlations were observed only among all psoriasis patients and those with mild psoriasis not receiving MS treatment. It is difficult to interpret these findings. Beyond the limited patient sample, other potential explanations could be that patients with psoriasis are more prone to having mild MS not requiring treatment, might be subject to misdiagnoses in medical records, or could receive an ICD-10 code for suspected MS that is later unconfirmed. However, it is unlikely that the lack of treatment for some MS patients indicates an uncertain diagnosis, given that MS typically follows a relapsing and remitting course, and not all MS patients receive treatment. Therefore, our results do not definitely suggest an increased likelihood of MS among patients with psoriasis, and larger epidemiological studies, incorporating treatment as an indicator for MS, are necessary for further clarification.

Obesity, hyperlipidemia, hypertension, diabetes, depression, angina pectoris and alcohol dependence are known comorbidities in psoriasi^18,49-51^ which is also confirmed by the results of this study. Smoking was also found to have a significant difference among psoriasis patients, aligning with prior studies that suggest a higher prevalence of smoking in psoriasis patients and its association with an increased risk of developing psoriasis.^19^

There are some limitations to this study. The list of included comorbid diseases is not comprehensive and it would have been interesting to include other important conditions as cardiovascular diseases and cancer. When interpreting the current data, it is important to bear in mind that the statistical model used in this study does not account for various crucial confounding factors, such as socio-economic status. It is recommended that these factors be taken into consideration in future studies. An ascertainment period was not established to assess the timing of comorbidities and their treatment in relation to the diagnosis of psoriasis. While this approach would have been ideal, the current study´s objective was to outline the prevalence of comorbid diseases and their treatments in individuals with psoriasis and without psoriasis, rather than establishing causal relationships. Retrospective studies are susceptible to miscoding and the possibility that participants may have received an inaccurate diagnosis. To mitigate this risk, this study employed specific criteria to validate the psoriasis diagnosis, requiring the diagnosis to be made once by a dermatologist or twice in primary care or by another clinic. Comorbidity diagnoses were validated by linking patient drug prescriptions to the diagnosis. When a patient received both a diagnosis and specific treatment, it reduces the likelihood that the initial diagnosis was incorrect. While some drugs may have been prescribed for indications other than the included comorbid condition, this may be a disadvantage of the study. There was no explicit indication of the specific condition for which a drug was prescribed. This leaves room for the possibility of biologics being prescribed for severe psoriasis, psoriatic arthritis, or other forms of arthritis, and potential overlap between these conditions. However, most medications are specific to a particular disease, such as insulin and oral antidiabetic drugs, which should not be prescribed to individuals who do not have diabetes. Distinguishing between these groups validates the medical record system and the reliability of the ICD coding.

Interestingly, our results did not reveal any evidence of undertreatment of comorbid conditions among patients with psoriasis. The proportion of patients with psoriasis receiving treatment for comorbidities was comparable to those without psoriasis . Nevertheless, we identified two noteworthy exceptions, one within the arthritis group and the other related to nicotine dependence. It appeared that all patients with psoriasis and mild psoriasis, are less likely to receive arthritis treatment when compared to patients without psoriasis. It is important to interpret these results cautiously, given the limitations previously mentioned, but they do indicate a potential undertreatment among psoriasis patients. Future research is needed to address this hypothesis. Notably, all patients with psoriasis and mild psoriasis were more likely to receive treatment for nicotine dependence, while those with severe psoriasis received more arthritis treatment. This implies that either physicians have increased awareness of comorbidities in psoriasis patients, or the more frequent healthcare contacts of these patients enhance the likelihood of detecting comorbidities.

When we adjusted the regression model to account for all comorbidities and lifestyle factors in a multivariable model, this mainly confirmed the increased association of comorbidities and psoriasis. Yet, it resulted in some significant changes. Specifically, the previously significant increased likelihood of receiving smoking cessation treatment vanished. These results potentially lend support to the hypothesis that the implication for physicians to support smoking cessation is probably higher in patients with multiple cardiovascular risk factors as obesity, diabetes and hypertension. Accounting for those factors led to an equal likelihood of receiving smoking cessation treatment. This could potentially serve as an explanation for the lack of significance observed for angina pectoris in the multivariable model. Adjusting for all the risk factors leading to angina pectoris, which are more abundant in psoriasis patients, eliminated the increased odds of having treated angina pectoris among psoriasis patients. The increased odds of Crohn´s disease among psoriasis patients disappeared in the multivariable model, except within the group of patients with severe psoriasis. This prompts the question of whether all the other comorbidities and lifestyle factors contribute to Crohn´s disease, while severe psoriasis alone can independently lead to Crohn´s disease.

This study provided valuable information about the prevalence of psoriasis and the associated odds of comorbidity. The findings highlighted that individuals with severe psoriasis had the highest odds of comorbidity. These results can help healthcare professionals become more aware of the potential comorbidities associated with psoriasis, leading to better healthcare and preventive measures. Although psoriasis is a common disease it is often undertreated^52^ which can increase the likelihood of comorbidity and mortality. Therefore, having a better understanding of potential comorbidities and risk factors can help in detecting and treating such conditions at an earlier stage, ultimately improving patient outcomes.

## Supplemental Material

Supplemental Material - Exploring comorbidity and pharmacological treatment patterns in psoriasis - a retrospective population-based cross-sectional study Click here for additional data file. Supplemental Material for Exploring comorbidity and pharmacological treatment patterns in psoriasis - a retrospective population-based cross-sectional study by Nelly Hamgård, Albert Duvetorp, Anna Hägg, Sandra Jerkovic Gullin, and Oliver Seifert in Journal of Multimorbidity and Comorbidity
