# Supplementary material for: Exploring comorbidity and pharmacological treatment patterns in psoriasis - a retrospective population-based cross-sectional study
Source: J Multimorb Comorb. 2023 Oct 30;13:26335565231212336. doi: 10.1177/26335565231212336 (PMC10617277; doi:10.1177/26335565231212336)
Supplement: Supplemental Material - Exploring comorbidity and pharmacological treatment patterns in psoriasis - a retrospective population-based cross-sectional study [file sj-pdf-1-cob-10.1177_26335565231212336.pdf]

Suppl. table 1. Frequency of comorbidity and related drug treatment in psoriasis patients and patients without psoriasis (treat+ = with treatment for comorbidity, treat- = without treatment).

Comorbidity	Patients without psoriasis (n=268,949) n (%)	All patients with psoriasis (n=4,587) n (%)	Patients with mild to moderate psoriasis (n=3,407) n (%)	Patients with severe psoriasis (n=1,180) n (%)
Arthritis treat+	2,370 (0.9)	318 (6.9)	36 (1.1)	282 (23.9)
Arthritis treat-	188 (0.07)	42 (0.9)	41 (1.2)	1 (0.08)
Nicotine dependence treat+	2,116 (0.8)	110 (2.4)	75 (2.2)	35 (2.9)
Nicotine dependence treat-	6,531 (2.4)	249 (5.4)	174 (5.1)	75 (6.3)
Diabetes treat+	16,096 (6.0)	591 (12.9)	438 (12.9)	153 (13.0)
Diabetes treat-	2,426 (0.9)	77 (1.7)	54 (1.6)	23 (1.9)
Depression treat+	29,524 (11.0)	774 (16.9)	553 (16.2)	221 (18.7)
Depression treat-	3,190 (1.2)	67 (1.5)	52 (1.5)	15 (1.3)
Alcohol dependence treat+	2,175 (0.8)	64 (1.4)	45 (1.3)	19 (1.6)
Alcohol dependence treat-	2,515 (0.9)	85 (1.9)	63 (1.8)	22 (1.9)
Obesity treat+	1,601 (0.6)	64 (1.4)	42 (1.2)	22 (1.9)
Obesity treat-	16,065 (6.0)	576 (12.6)	407 (11.9)	169 (14.3)
Crohn disease treat+	932 (0.3)	24 (0.5)	10 (0.3)	14 (1.2)

Crohn disease treat-	165 (0.1)	4 (0.1)	3 (0.1)	1 (0.1)
Angina pectoris treat+	6,878 (2.6)	218 (4.8)	161 (4.7)	57 (4.8)
Angina pectoris treat-	929 (0.3)	37 (0.8)	24 (0.7)	13 (1.1)
Hypertension treat+	60,819 (22.6)	1,840 (40.1)	1,345 (39.5)	495 (41.9)
Hypertension treat-	1,800 (0.7)	49 (1.1)	32 (0.9)	17 (1.4)
Hyperlipidemia treat+	29,868 (11.1)	986 (21.5)	746 (21.9)	240 (20.3)
Hyperlipidemia treat-	1,448 (0.5)	41 (0.9)	26 (0.8)	15 (1.3)
Multiple sclerosis treat+	224 (0.1)	7 (0.2)	6 (0.2)	1 (0.1)
Multiple sclerosis treat-	419 (0.2)	14 (0.3)	13 (0.4)	1 (0.1)
